# Incidence and molecular epidemiology of hepatitis C virus reinfection in prisons in Catalonia, Spain (Re-HCV study)

**DOI:** 10.1038/s41598-023-42701-1

**Published:** 2023-09-25

**Authors:** Verónica Saludes, Antoni E. Bordoy, Elena Yela, Elisabet Turú, Anna Not, Evelin López-Corbeto, Laia Egea-Cortés, Fernando González-Candelas, Jordi Casabona, Group for the Study and Control of Infectious Diseases in Prison (GRUMIP), Núria Teixidó, Anna Sastre, Ana Ruíz, Carlos Gallego, Carlos Touzón, Concepció Solé, Ramón Planella, Elisa Vaz, Rafael A. Guerrero, Andrés Marco, Elisa Martró

**Affiliations:** 1 Microbiology Department, Northern Metropolitan Clinical Laboratory, Germans Trias i Pujol Research Institute and Hospital (IGTP), Crta. del Canyet S/N, 08916 Badalona, Barcelona Spain; 2 Consortium for Biomedical Research in Epidemiology and Public Health (CIBERESP), Instituto de Salud Carlos III, Madrid, Spain; 3 Brians-1 Prison Health Services, Sant Esteve Ses Rovires, Barcelona, Spain; 4 Prison Health Programme, Catalan Institute of Health (ICS), Barcelona, Spain; 5 Centre for Epidemiological Studies On Sexually Transmitted Infections and HIV/AIDS of Catalonia (CEEISCAT), Public Health Agency of Catalonia (ASPCAT), Badalona, Spain; 6 Joint Research Unit Infection and Public Health FISABIO-University of Valencia I2SysBio, Valencia, Spain; 7 EAPP Sant Esteve Sesrovires-1, Barcelona, Spain; 8 EAPP Sant Esteve Sesrovires-2, Barcelona, Spain; 9 EAPP La Roca del Vallés-1, Barcelona, Spain; 10 EAPP Sant Joan de Vilatorrada, Barcelona, Spain; 11 EAPP Figueres, Girona, Spain; 12 EAPP Lleida, Lleida, Spain; 13 EAPP Tarragona, Tarragona, Spain

**Keywords:** Infectious-disease diagnostics, Molecular medicine

## Abstract

Hepatitis C virus (HCV) reinfection may hamper HCV elimination in prisons. We aimed to (i) determine the reinfection rate in people treated for HCV in Catalan prisons, (ii) measure reinfection in people entering prisons, and (iii) characterize the molecular epidemiology of HCV in prisons and people who inject drugs (PWID) in the community. Re-HCV was a prospective study in eight prisons (2019–2020) including two groups: (1) people cured with treatment in prison and followed-up every 6 months, and (2) people testing HCV-RNA positive at incarceration. Bio-behavioral data were collected. HCV isolates were sequenced and phylogenetically analyzed with those of PWID in the community. Reinfection follow-up after treatment was achieved in 97 individuals (103.05 person-years). Two reinfections were detected, resulting in an incidence ≤ 10/100 person-years. Among people entering prison, 2% (359/17,732) were viremic, of which 334 (93.0%) were included, and 44 (13.5%) presented with reinfection (84.7% being PWID). Frequently, HCV isolates in prisons and PWID in the community were phylogenetically related. Although HCV reinfection is low after treatment, it is common in people entering Catalan prisons. To maintain a low HCV prevalence in prisons, harm-reduction services and test-and-treat programs for PWID should be strengthened both inside and outside prisons.

## Introduction

Spain is among the countries on track to reach the absolute HCV elimination targets set by the World Health Organization (WHO), that is, an absolute annual HCV incidence of ≤ 5 cases per 100,000 persons and ≤ 2 cases per 100 people who inject drugs (PWID), and an HCV-related annual mortality rate of ≤ 2 deaths per 100,000 persons^1^.

People in prisons are considered a key population to be screened and treated for HCV infection in the WHO strategy for HCV elimination as well as by the Spanish national and regional Hepatitis C Plans (including that from Catalonia)^1–3^. The prison setting enables access to populations at high risk of HCV infection, such as PWID, who additionally have suboptimal engagement with mainstream health services outside prison. Additionally, people in prisons are a dynamic population with high mobility between the prisons and with the community. Therefore, curing hepatitis C in this population has a significant impact on public health^4^.

Currently, 7913 individuals (93.5% men and 47.9% foreigners) are incarcerated within the Penitentiary System of Catalonia, an autonomous region in Spain with a population of 7.5 million people. The stay in Catalan prisons offers an opportunity to detect, prevent, and treat hepatitis C, as the whole range of existing services aiming at eliminating HCV infection is offered according to WHO recommendations^5^: (i) voluntary HCV screening upon entering prison (coverage of 84%) and periodic screening if risk behaviors for HCV infection are maintained; (ii) use of directly acting antivirals (DAA) therapy for hepatitis C and periodic HCV-RNA screening after cure to detect reinfection; (iii) opioid substitution therapy (OST) and needle and syringe programs (NSP); and (iv) educational activities on HCV prevention. Furthermore, the continuity of HCV care and treatment is guaranteed by the figure of a liaison nurse after release.

Since the implementation of DAA-based therapy against HCV infection in 2015 in Catalan prisons, more than a thousand people have been treated during imprisonment and have achieved a sustained virological response (SVR)^6^. Unfortunately, when risk behaviors for HCV infection are maintained over time, reinfection can occur one or more times after cure, as the immunity generated against HCV infection is not protective. In a study performed by Marco et al. in a cohort of people treated in the Catalan penitentiary system between 2002 and 2016, HCV-RNA was assessed once a year according to the existing guidelines at that time^7^, and a relatively low reinfection rate of 3.9 per 100 person-years was observed in PWID^8^. However, higher reinfection rates post-SVR of 31 and 16.7 cases per 100 person-years among PWID in the community have been reported in Catalonia and Madrid, respectively^9,10^, and it is well known that PWID are at a higher risk of imprisonment during their lifetime^11^. Nevertheless, the proportion of HCV reinfection among people entering prisons has not yet been determined.

Given that people in prisons constitute a dynamic population, it is necessary to diagnose and treat reinfections as soon as possible to avoid the spread of the virus and identify the determinants of these reinfections. Therefore, in the present study, we sought to re-evaluate HCV reinfection post-SVR in the Catalan Penitentiary System using frequent HCV-RNA testing intervals (every 6 months) together with sequence analyses of HCV isolates circulating within the prison setting and in the community. Specifically, we aimed to (i) determine the current HCV reinfection rate after cure by therapy (post-SVR) and its determinants in people treated in Catalan prisons, (ii) assess the proportion of HCV reinfection in people entering prisons, and (iii) characterize the molecular epidemiology of the HCV epidemic in the prison setting in relation to the community.

## Methods

### Study design and participants

Re-HCV was a prospective, observational, multicenter study of HCV reinfection performed over two years (January 2019–December 2020) in eight Catalan prisons with medical services able to administer HCV treatment. The characteristics of people in prison from each penitentiary center included in the study were as follows: Brians-1, men on preventive detention and sentenced women; Brians-2, sentenced men; Lledoners, sentenced men; Quatre Camins i Joves, sentenced men, and a separate facility for young men (aged 18 to 21); Dones, women on preventive detention; Mas d'Enric, men and women, sentenced and on preventive detention; Ponent, men and women, sentenced and on preventive detention; Puig de les Basses, men and women, sentenced and on preventive detention.

Prison medical and nursing staff informed individuals over 18 years of age about the study, gave them an information sheet, and asked them to participate by signing an informed consent form if they fulfilled the following criteria to enter into one of the two defined study groups.

#### Reinfection follow-up group

This group included people cured by therapy in prison and followed up for reinfection at 6-month intervals. All individuals who achieved SVR at week 12 (SVR12) after DAA treatment while in prison from July 2018 to July 2020, were included in the study at the 6 months post-SVR time-point and were subsequently followed up every 6 months (at 12, 18, and 24 months post-SVR). HCV reinfection was defined as a secondary infection following a primary infection cured by DAA treatment, with SVR confirmed by a negative HCV-RNA test. Participants who were released from prison were considered lost to follow-up.

#### Viremic group

This study group included all individuals in prison with viremic HCV infection detected at the time of imprisonment over the entire study period. Individuals in this group who initiated DAA treatment and achieved cure (SVR12) were candidates for subsequent inclusion in the reinfection follow-up group. In this group, HCV reinfection was defined as a secondary infection following a primary infection cured either spontaneously or by DAA treatment (SVR confirmed by a negative HCV-RNA test result).

### Clinical specimens

Venous blood samples were obtained, as described below, by the nursing staff at each prison to assess the presence of HCV viremia. The samples were shipped to the reference territorial laboratories assigned to each prison for HCV-RNA testing.

#### Reinfection follow-up group

Blood samples were obtained at 6, 12, 18, or 24 months after SVR as long as the participants were still incarcerated at these time points.

#### Viremic group

Blood samples were obtained at incarceration as part of the routine admission process.

When HCV-RNA was detected by the reference territorial laboratories at any of the time points defined above, a frozen EDTA-plasma aliquot was shipped to the coordinator laboratory for HCV sequencing.

### HCV sequencing and phylogenetic analyses

To characterize the molecular epidemiology of the HCV epidemic in the prison setting in relation to the community, isolates from participants in the viremic group were sequenced and phylogenetically analyzed together with isolates circulating in the community. The latter were obtained from 291 PWID who attended four harm reduction services (HRS) in Barcelona province, caring for half of the PWID population in Catalonia from the HepC*detect* II study between May 2016 and February 2019^12,13^. The methods used, including total RNA extraction, retrotranscription, PCR amplification, Sanger sequencing, and phylogenetic and clustering analyses of the HCV NS5B region, are described in the Supplementary material S1.

### Epidemiological data collection

Sociodemographic and behavioral information was collected through a brief questionnaire (Questionnaire A) completed by participants, which included questions about drug use and sexual practices both inside and outside the prison. In parallel, the prison healthcare personnel completed a questionnaire (Questionnaire B) with information related to the treatment of hepatitis C, as well as sociodemographic characteristics and clinical-therapeutic data of the participants. In the reinfection follow-up group, both questionnaires were completed at enrollment (6 months post-SVR) with questions about the treatment period and at each follow-up time point (12, 18, and 24 months post-SVR) in order to assess behavioral changes over time after cure and their possible association with reinfection. In the viremic group, both questionnaires were completed at the time of incarceration, with questions about their entire lives.

### Statistical analyses

For quantitative variables, we used measures of central tendency and dispersion (medians and 95% interquartile ranges). For qualitative variables, we calculated the absolute frequencies and percentages. Bivariate analysis was carried out using the Mann–Whitney U test to compare medians and chi-square tests to compare categorical differences. In the reinfection follow-up group, the statistical analyses consisted of determining the reinfection rate after cure by DAA therapy in prison and comparing the characteristics of the reinfected and non-reinfected cases. The reinfection incidence rate was calculated for the population as cases per 100 person-years of follow-up after censoring at the last negative HCV RNA test or at the midpoint between the last negative and first positive HCV RNA test dates for those with reinfection. The proportions of clustered isolates from the Re-HCV and HepC*detect* II studies were calculated and compared using Fisher’s exact test, *p*-values were adjusted using the Benjamini and Hochberg method^14^ to decrease the false discovery rate. All analyses were performed using R version 4.0.3.

### Ethics declarations

This study was approved by the institutional Ethics Board at Germans Trias i Pujol University Hospital (PI-18-247, January 11, 2019), and the participants provided written informed consent. The study was conducted in accordance with the requirements established by the current legislation.

## Results

### Characteristics of the study population

**Figure 1:**
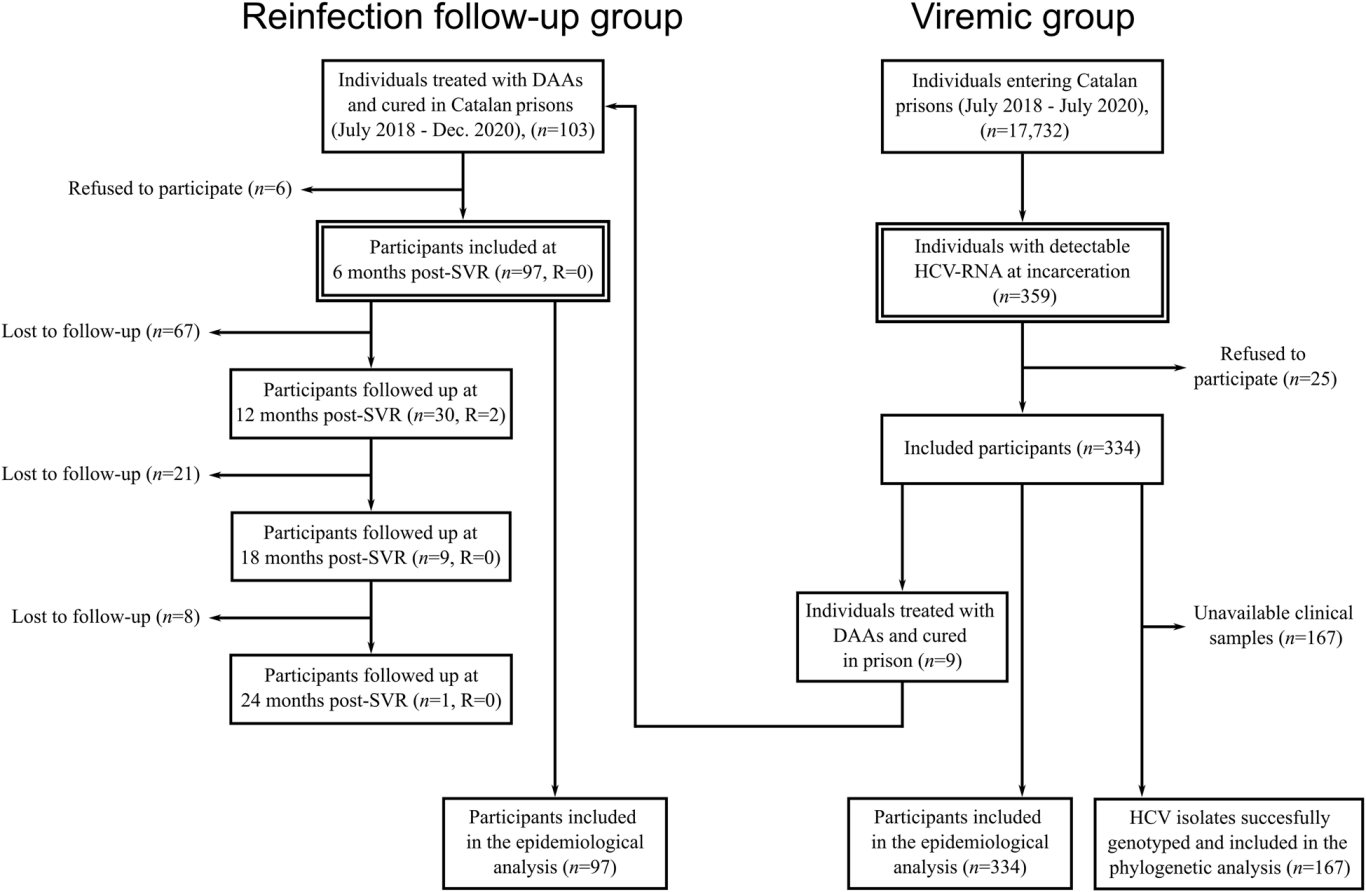
Flowchart of participants included in the reinfection follow-up and viremic study groups. *R* reinfection.

Overall, 422 individuals were included in this study (Fig. 1). In the reinfection follow-up group, study inclusion and reinfection follow-up were performed in 97 individuals treated with DAAs in Catalan prisons from January 2018 to December 2019, while six individuals refused to participate (6/103, 5.8%). The reason for not participating was refusing the blood draw required for reinfection assessment in 4/6 (66.7%) cases; one individual was transferred out of the prison, and the last one did not provide any reason.

Among the 17,732 people who entered the Catalan penitentiary system and were tested for HCV-RNA during the study period (Fig. 1), 2% (n = 359) were viremic and 93.0% (n = 334) agreed to participate in the study (viremic group). Reasons for not participating were provided in 12/25 (48%) cases and were highly diverse. Nine individuals in the viremic group were included in the reinfection follow-up group after treatment and SVR achievement (9/97).

The main characteristics of the participants at the time of inclusion in the study for each study group are shown in Table 1. Briefly, most individuals were men, with a median age of 41 years. Foreign-born participants accounted for 29% and 46% of the reinfection follow-up and viremic groups, respectively. Approximately a quarter of the participants were HIV-positive, and a similar proportion presented with a psychiatric disorder. Most people in the Catalan prisons who participated in the present study reported drug injection at least once during their lives (> 80%), and 28% of the viremic group participants reported drug injection during previous incarcerations. Intravenous drug use during hepatitis C treatment was reported by one third of the reinfection follow-up group participants. Additionally, 86.5% and 75.3% of the reinfection follow-up group and viremic group participants, respectively, had previously been in prison.

**Table 1:** Characteristics of the Re-HCV study participants (N = 422)** at study inclusion.

Characteristics	Reinfection follow-up group (*n* = 97) n (%)	Viremic group (*n* = 334) n (%)
Age, median (range)	44 (30–59)	41 (26–57)
Gender		
Male	86 (88.7)	311 (93.1)
Female	10 (10.3)	23 (6.9)
Foreign origin	28 (28.9)	153 (46.1)
HIV infection	23 (23.7)	103 (30.8)
Psychiatric disorder	24 (24.7)	76 (23.0)
Educational attainment > primary	56 (63.6)	201 (60.7)
Homeless during the 6 months before incarceration	53 (58.2)	61 (18.5)
Previously incarcerated	77 (86.5)	250 (75.3)
Sexual orientation		
Heterosexual	85 (95.5)	319 (96.1)
Homosexual	1 (1.1)	5 (1.5)
Bisexual	3 (3.4)	8 (2.4)
Intravenous drug use at least once during live	74 (82.2)	283 (84.7)
Intravenous drug use in prison	–	80 (28.3)
Intravenous drug use during hepatitis C treatment	30 (31.3)	–
Intravenous drug use during hepatitis C treatment while in prison	12 (40.0)	–
Sharing of needles*	16 (16.7)	140 (41.9)
Sharing of needles in prison*	9 (56.3)	46 (33.6)
Sharing of syringes*	17 (17.9)	145 (43.5)
Sharing of syringes in prison*	10 (55.6)	45 (32.4)
Sharing of other injection equipment*	19 (19.8)	169 (50.6)
Sharing of other injection equipment in prison*	9 (47.7)	47 (28.5)
Practicing front-backloading*	11 (11.5)	102 (30.5)
Practicing front-backloading in prison*	5 (45.5)	27 (27.6)
Sharing drugs already prepared to inject*	17 (17.7)	174 (52.3)
Sharing drugs already prepared to inject in prison*	9 (52.9)	45 (26.5)
Cocaine snorting*	45 (46.9)	288 (86.2)
Cocaine snorting in prison*	28 (65.1)	117 (41.2)
Sharing cocaine snorting straw*	34 (35.8)	215 (64.8)
Sharing cocaine snorting straw in prison*	20 (60.6)	89 (42.0)
Piercing or tattooing*	33 (34.4)	250 (74.9)
Piercing or tattooing in prison*	18 (54.4)	119 (48.0)
Unprotected sex*	21 (21.9)	165 (49.4)
Unprotected sex in prison*	5 (23.8)	26 (16.0)
Currently in treatment for drug addiction*	62 (64.6)	164 (49.2)
Released, on prison furlough, or released under surveillance during the present sentence*	23 (24.2)	33 (9.9)

*These questions referred to the hepatitis C treatment period for the reinfection follow-up group, and to at any time in life for the viremic group.

**Nine individuals of the viremic group were candidates to be subsequently included in the reinfection follow-up group after treatment and SVR achievement.

Baseline HCV genotypes in the reinfection follow-up group participants, as reported in the epidemiological questionnaire were as follows: genotype 1 (53.6%), 3 (23.7%), 4 (17.5%), 2 (1.0%), mixed infection (3.1%), and undetermined (1.0%). HCV genotypes in the viremic group participants were determined by sequencing and phylogenetic analysis, as detailed below.

### Observed HCV reinfection in individuals treated with DAAs in Catalan prisons (reinfection follow-up group)

Among the 97 participants included in this group at 6 months post-SVR, 30.9% (30/97), 9.3% (9/97), and 1.0% (1/97) were followed-up at 12, 18, and 24 months post-SVR respectively, resulting in a mean follow up time of 8.5 months post-SVR. Most losses to follow up (69.1%, 67/97) occurred during the first 6 months after study inclusion (i.e., within the 12–18 months post-SVR period). The main reasons for loss to follow-up were release from prison due to sentences or preventive detention ending, or premature release due to the COVID-19 pandemic.

**Figure 2:**
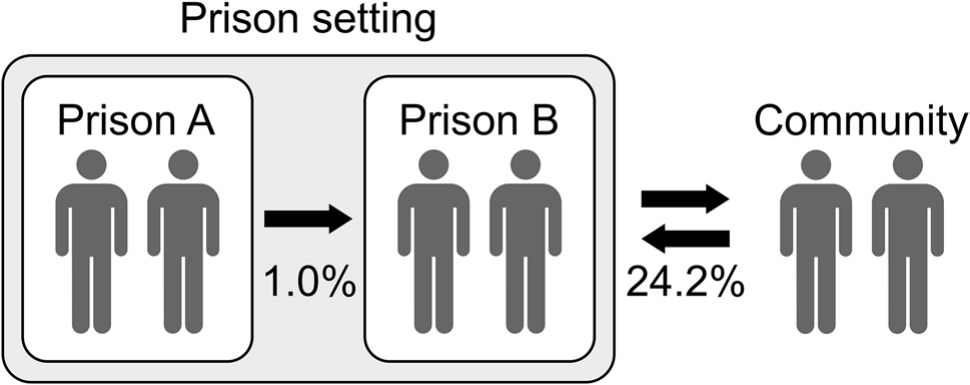
Schematic representation of mobility of participants in the reinfection follow-up group between prisons and with the community over the study period.

The mobility of the reinfection follow-up group participants between the prisons and the community is shown in Fig. 2. Movement between prisons took place at least once in 1.0% (1/97) of the participants during follow-up. Additionally, 24.2% (23/95) of the participants were released and later incarcerated again, released under surveillance during incarceration, or on a prison furlough.

During the 103.05 person-years of follow-up, two cases of reinfection were identified according to clinical records, both at 12 months post-SVR, which translates into an incidence of reinfection of 2 per 100 person-years (95% CI 0–7). The low number of detected reinfection cases prevented the identification of the determinants of reinfection in this group. However, it is important to note that these two cases reported intravenous drug use at least once during their lives, as well as other risky behaviors associated with HCV infection. Both cases reported more than three previous incarcerations and risk behaviors were maintained while in prison. Furthermore, one individual was co-infected with HIV. Both participants were released between the end of treatment and detection of reinfection at incarceration (12 months post-SVR). Both cases were then treated and cured while in prison.

### Characteristics of viremic people entering prisons (viremic group)

Among the viremic participants incarcerated during the study period (*n* = 334), 84.7% (283/334) were classified as primary infections, 13.2% (44/334) as reinfections after successful antiviral treatment, and 2.1% (7/334) as treatment failures according to the available clinical records.

The characteristics of participants with reinfection and primary infection are shown in Table 2 (the low number of treatment failures precluded their characterization). Briefly, HIV infection, homelessness, and having been in prison more than three times were significantly more frequent in those with HCV reinfection than in those with primary infection. Regarding risk behaviors, injecting drugs, sharing syringes and needles, and unprotected sex were also more frequent in those reinfected, either at some point in life or in prison (during previous imprisonments). Additionally, HCV subtype 3a infection was detected more frequently among reinfected individuals at incarceration than other genotypes (Supplementary Fig. S1).

**Table 2:** Comparison of the characteristics of participants presenting with primary infection or reinfection at incarceration (viremic group).

Characteristics	Primary infection (n = 283) n (%)	Reinfection (n = 44) n (%)	*p*-value
Age, median (95% interquartile range)	40 (25.0–57.0)	41 (29.0–53.0)	n.s
Gender			
Male	261 (92.2)	43 (97.7)	n.s
Female	22 (7.8)	1 (2.3)	
Foreign origin	134 (47.5)	18 (41.9)	n.s
HIV infection	80 (28.3)	21 (47.7)	0.009
Psychiatric disorder	66 (23.6)	7 (15.9)	n.s
Educational attainment > primary	174 (62.1)	24 (54.6)	n.s
Homeless*	38 (13.7)	16 (36.4)	< 0.001
Previously incarcerated (≥ 3 times)	145 (51.6)	33 (75.0)	0.013
Sexual orientation			
Heterosexual	270 (96.1)	43 (97.7)	n.s
Homosexual	5 (1.8)	0 (0)	
Bisexual	6 (2.1)	1 (2.3)	
Intravenous drug use at least once during live	235 (83.0)	43 (97.7)	0.011
Intravenous drug use in prison	58 (24.7)	19 (44.2)	0.009
Sharing of needles at least once during live	109 (38.5)	28 (63.6)	0.002
Sharing of needles in prison	29 (27.1)	16 (59.3)	0.002
Sharing of syringes at least once during live	112 (39.7)	30 (68.2)	< 0.001
Sharing of syringes in prison	29 (27.1)	15 (51.7)	0.012
Sharing of other injection equipment at least once during live	136 (48.1)	28 (63.6)	0.055
Sharing of other injection equipment in prison	33 (24.8)	13 (46.4)	0.021
Practicing front-backloading at least once during live	77 (27.2)	22 (50.0)	0.002
Practicing front-backloading in prison	18 (24.7)	8 (36.4)	n.s
Sharing drugs already prepared to inject at least once during live	138 (48.9)	32 (72.7)	0.003
Sharing drugs already prepared to inject in prison	32 (23.7)	12 (37.5)	n.s
Cocaine snorting at least once during live	241 (85.2)	40 (90.9)	n.s
Cocaine snorting in prison	93 (39.1)	21 (53.9)	0.082
Sharing cocaine snorting straw at least once during live	178 (63.4)	31 (70.5)	n.s
Sharing cocaine snorting straw in prison	68 (38.9)	17 (54.8)	n.s
Piercing or tattooing at least once during live	209 (73.9)	37 (84.1)	n.s
Piercing or tattooing in prison	93 (44.9)	23 (62.2)	0.053
Unprotected sex at least once during live	132 (46.6)	28 (63.6)	0.036
Unprotected sex in prison	16 (12.4)	9 (32.1)	0.01
Currently in treatment for drug addiction	134 (47.5)	29 (65.9)	0.061
Released, on prison furlough, or released under surveillance during the present sentence	27 (9.5)	4 (9.1)	n.s

*Previous 6 months before incarceration.

*n.s.* not significant.

### Molecular epidemiology analysis evidenced that HCV isolates detected inside and outside the prison setting were commonly related

Due to COVID-19 related constraints, clinical samples could not be recovered from the reference laboratories for 167 (50%) viremic group participants. Therefore, 167 HCV isolates were genotyped by sequencing and phylogenetic analysis of the viremic group participants. The distribution of HCV genotypes detected in individuals entering Catalan prisons is shown in Supplementary Fig. S1. Overall, the most frequently detected genotype was 1a (36.5%, 61/167) followed by 3a (27.5%, 46/167).

**Figure 3:**
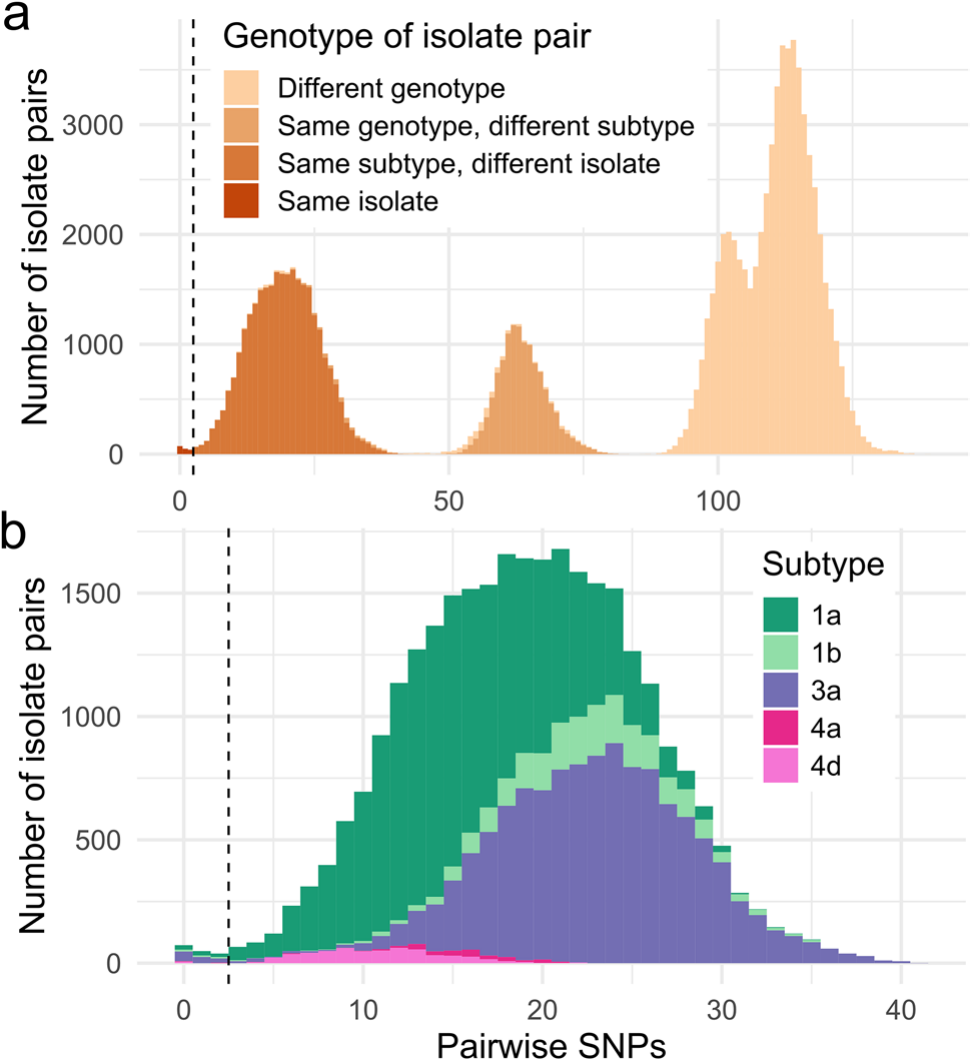
Stacked histogram of pairwise SNP differences between HCV sequences. (**a**) Pairwise SNP differences between sequences obtained from Re-HCV and Hep*Cdetect* II studies (*n* = 458). (**b**) Pairwise SNP differences between HCV isolate pairs of selected subtypes. In both panels, the black dashed line indicates a pairwise SNP threshold of two.

To identify recent transmission events involving HCV isolates detected in people in prisons, a pairwise SNP distance cut-off of 2 (within the NS5B fragment sequenced) was set based on the distribution of pairwise SNP differences between pairs of sequences of the same subtype (Fig. 3). The cut-off value corresponds to 98.4% for the cumulative distribution function of the Poisson distribution with λ = 0.51, observed for low pairwise SNP values. As expected, the mode of such distribution, ⌊λ⌋ = 0, was lower than the estimated number of SNPs (2.3) in a period of five years, which was the maximum timespan during which transmission between Re-HCV and HepC*detect* II study participants could have taken place. Sequence pairs below this cut-off were considered to share the most recent common ancestor during the previous five years at most, thus providing strong evidence of recent transmission (either directly or from shared sources). Transmission clusters fulfilling this cut-off (supported by a monophyletic origin with a bootstrap value > 90%) were identified within each HCV subtype and are depicted in Supplementary Fig. S2 and described in Table 3. Overall, 25 recent transmission clusters were identified containing at least one isolate from the Re-HCV study; corresponding to 24.0% (40/167) of sequenced isolates from viremic group participants. Identified clusters also involved 7.9% (23/291) of HepC*detect* II study participants (HRS setting). Eight (32.0%) clusters contained exclusively Re-HCV study participants and had a median size of two (range 2–4); the rest of the clusters (*n* = 17) showed the same median size and a similar range (2–5), but contained at least one participant from Re-HCV and one from HepC*detect* II. Among the latter, the sequences found in people in prison and the HRS were identical in seven clusters. There were 10 clusters containing more than one Re-HCV isolate, of which five contained isolates from different prisons, and six included identical sequences from 12 different individuals. Overall, 12.6% (21/167) of the sequences collected from people in prison belonged to a transmission cluster involving sequences from the community. The percentage of clustered sequences across different HCV subtypes varied from 0 (genotype 2 and subtype 6a, 0/1) to 45.2% (subtype 1b, 14/31), whereas this percentage was higher in reinfection cases (36.8%, 7/19) than in treatment failure (33.3%, 1/3) and primary infection cases (22.1%, 32/145) (Table 3). However, the differences in the frequency of clustered Re-HCV samples among different HCV subtypes as well as among different infection types were not statistically significant.

**Table 3:** Number of sequences involved in recent transmission clusters according to HCV subtype and infection type among viremic group participants.

HCV subtype	Patient type			Sequences in clusters^1^	No. of sequences^2^	Clustering ratio^3^
	Primary infection	Treatment failure	Reinfection			
1a	10 (6.0%)	–	3 (1.8%)	13 (7.8%)	61 (36.5%)	21.3
1b	12 (7.2%)	–	2 (1.2%)	14 (8.4%)	31 (18.6%)	45.2
2	–	–	–	–	1 (0.6%)	–
3a	5 (3.0%)	1 (0.6%)	2 (1.2%)	8 (4.8%)	46 (27.5%)	17.4
4a	2 (1.2%)	–	–	2 (1.2%)	10 (6.0%)	20.0
4d	3 (1.8%)	–	–	3 (1.8%)	17 (10.2%)	17.6
6a	–	–	–	–	1 (0.6%)	–
Sequences in clusters	32 (19.2%)	1 (0.6%)	7 (4.2%)	40 (24.0%)	–	
No. of sequences	145 (86.8%)	3 (1.8%)	19 (11.4%)	–	167 (100%)	
Clustering ratio	22.1	33.3	36.8			

^1^No. of sequences from each subtype in clusters, from the total number of sequences.

^2^No. of sequences from each subtype, from the total number of sequences.

^3^No. of sequences in cluster, from the total number of sequences.

## Discussion

This study shows that after over a decade of HCV treatment with DAAs in Catalan prisons, the reinfection rate is low in this setting. However, viremic HCV infection in people entering prisons is frequently due to reinfection, especially among those most vulnerable (HIV-infected and homeless individuals) and maintaining risk behaviors inside and outside prison. This may hamper HCV elimination both in the prison setting and in the community (i.e., harm reduction centers), given the high mobility of individuals at risk between these two settings.

Most people in the Catalan prisons who participated in the present study reported drug injection at least once during their lives (> 80%), and 28% of the participants reported drug injection during previous incarcerations. This is in agreement with the fact that injection drug use is the main contributor to both prevalent and incident HCV infections among people in prisons globally^15^. In line with this, in the HepC*detect* II study carried out on PWID who accessed HRS in the province of Barcelona, a 58.5% prevalence of viremic HCV infection was observed, 64% of the participants reported having been in prison at least once and 27% reported injecting drugs while in prison^16^. Tattooing, piercing, and high-risk sexual behaviors have also been described as HCV-associated factors among people in prisons^11^, and the self-reported proportions of these behaviors cannot be considered negligible in the study population presented here. Additionally, about a quarter of the participants were HIV-infected, which has been associated with a higher risk of HCV reinfection among prisoners^6,8^.

Despite the high prevalence of these risk factors in the reinfection follow-up group, only two cases (2%) of HCV reinfection after achieving SVR with DAA treatment in the Catalan Penitentiary System were detected over the study period, leading to a low reinfection rate (< 10 per 100 person-years). Both cases occurred in individuals who reported intravenous drug use as well as other risk behaviors associated with HCV infection. These results are in agreement with a previous study by Marco et al. in the same setting that also found an incidence of HCV reinfection of < 10 per 100 person-years (3.62; 95% CI 1.28–7.74) from 2015 to 2016; all reinfections were detected in individuals who reported injecting drug use at least once during their lives^8^. However, Marco et al. might have underestimated the reinfection rate using a 12-month testing interval, as spontaneously cleared infections within this 12 month intervals would have been missed. In a previous study, up to 52% of PWID who achieved SVR and got reinfected, spontaneously cleared HCV-RNA six months after reinfection^17^. Thus, within the context of the Re-HCV study, monitoring of HCV reinfection by HCV RNA testing was implemented every six months post-SVR for the first time in the Catalan Penitentiary System^5^, in agreement with the available recommendations^18^. In our study, reinfection between the end of treatment and SVR was not evaluated; nevertheless, a low rate of reinfection within this period would be expected, according to previous data^8^. SVR rates in the Catalan prison setting are ≥ 95%, and the absence of SVR is more frequent in HCV genotype 3 infection, liver cirrhosis, low treatment adherence, and/or interruption of treatment due to prison release^6^. Cuadrado et al. reported no reinfections in the JAILFREE-C trial in a prison in Cantabria (Spain) between 2016 and 2017, either before SVR (three virological failures were observed) or after SVR (with a median follow-up time of 12 months; range 5.5–14)^19^. By contrast, the few studies performed so far on HCV reinfection in people treated in non-Spanish prisons have observed elevated reinfection rates, mainly among those with maintained injection drug use and sharing of associated paraphernalia after cure: 12.5 cases per 100 person-years (95% CI 7.9–19.8) in the STOP-C trial performed in Australia between 2014 and 2019^20^, 14.3 per 100 person-years (95% CI 11.1–18.5) in a large study performed in Scotland between 2000 and 2018^21^, and 40.6 per 100 person-years in England between 2016 and 2019^22^.

The observed differences in reinfection rates among people treated in prisons across different countries and regions may be due not only to the local prevalence of viremic HCV infection among people in prisons, but also to the approaches adopted in each country to reach the goal of HCV elimination in prisons, among other factors. It is well recognized that access to harm-reduction programs within prisons substantially reduces the risk of HCV reinfection^23^. However, people in prisons in Australia and the United Kingdom have access to OST but not to NSP^4,24^. In Spain, prisons from Catalonia and Cantabria offer the whole range of services aimed at eliminating HCV infection^5,19^, which, in combination with the continuum of care program contributes to the reduction of the HCV reinfection rate observed among people treated with DAAs in these prisons. Additionally, the availability of these services in Catalan prisons has led to a progressive reduction in the prevalence of viremic HCV infection in this setting, from 10% in 2015 to 0.6% in the first quarter of 2023^25^, which is similar to the prevalence of viremic HCV infection in the general population in Catalonia (0.49%)^26^. Since both participants with detected reinfection in the present study were released between the end of treatment and the time of reinfection detection (12 months post-SVR when they were re-incarcerated), we hypothesized that they were possibly reinfected outside the prison setting after their release. An increased risk of HCV acquisition in the first period after its release from prison has been described^11^. Furthermore, the persistence of high-risk behaviors among PWID during a prison furlough or after release from imprisonment is well known^27^.

Despite the low HCV reinfection rate in people treated with DAAs in Catalan prisons, HCV reinfection was common (13.2%) among people entering Catalan prisons. Specifically, socioeconomic factors (homelessness and having previously been in prison), risk behaviors practiced during lifetime or while in prison (injecting drugs, sharing syringes and needles, and unprotected sex), and HIV infection were more frequently detected in individuals entering prison with HCV reinfection than in those with primary infection, indicating that this population shows a highly vulnerable and risky behavioral profile for HCV infection. Prevention strategies in Catalan prisons, including access to OST, NSP, and condoms, have led to a decrease in HCV infection during incarceration. However, it should be borne in mind that almost half of the participants were migrants, and information on the location of previous imprisonments was not available; therefore, prevention strategies available during their preceding imprisonments were unknown. In agreement with the obtained results, previous studies indicate that risk behaviors for HCV infection often continue during and after antiviral treatment in PWID, putting them at risk of reinfection, which compromises the individual and population-level benefits of HCV treatment-as-prevention strategies. In fact, a much higher reinfection rate (31 cases per 100 person-years) has been reported by Lens et al. among PWID diagnosed and treated in a HRS in Catalonia, mainly in association with HIV infection and daily drug injection^9^. Although most of the individuals had previously been in prison, this was not identified as an independent predictive factor for reinfection. Moreover, overall reinfection rates post-SVR in PWID of 3.8–6.2 cases per 100 person-years have been reported in a meta-analysis^23^. Altogether, these data highlight the need for continued HCV-RNA screening after therapy, early reinfection diagnosis, and access to re-treatment both in prisons and the community to prevent onward transmission. In addition, risk-reduction strategies and education should be provided to high-risk populations who initiate treatment to prevent reinfection.

In the present study, close epidemiological relationships were identified between HCV isolates from people entering Catalan prisons with viremic HCV infection and from PWID accessing community HRS in Barcelona, as well as between HCV isolates from people located in different prisons. Importantly, about a quarter of the Re-HCV study participants belonged to transmission clusters. This phylogenetic analysis reflected the dynamic nature of these populations, in accordance with previous evidence in European prisons^11^. In agreement with this, more than 75% of participants had been in prison before, and at least a quarter of the reinfection follow-up group participants were released and later incarcerated again, released on prison furlough, or released under surveillance during incarceration. Consequently, it is necessary to guarantee the continuity of care across the prison-community interface and to retreat HCV reinfections as soon as possible to avoid the spread of the virus in both the prison and the community^23^. Currently, in Catalan prisons, the liaison nurse links people released from prison to HCV care in the community^5,28^. This strategy is likely to improve the previously limited linkage to mainstream HCV healthcare services and treatment among active PWID accessing the HRS network of Catalonia, especially in migrant PWID^29^. Additionally, Lens et al. recently demonstrated that PWID can be successfully treated through an externalized hepatology outpatient clinic located in a HRS in Barcelona^9^. Altogether, these observations support the need to expand decentralization and access to early diagnosis and treatment of hepatitis C reinfection among PWID in the community in locations frequented by PWID. Otherwise, HCV elimination will not be achieved in this group.

This study has been challenging in many aspects given its multicentric nature (eight prisons involved with different medical teams) and the fact that many participants were on preventive detention or had short sentences. The latter was also due to the fact that 17% of individuals were released from Catalan prisons during the first wave of the pandemic to prevent the spread of SARS-CoV-2^30^, leading to a sample size and follow-up time of the reinfection group lower than expected. Consequently, a wide 95%CI was obtained for the reinfection rate among people treated with DAAs in Catalan prisons, and identification of the determinants of reinfection was not possible. Additionally, plasma samples for HCV sequencing could only be obtained from 46.7% of the viremic participants because of the great work overload caused by the large-scale SARS-CoV-2 diagnostic activity. This fact also precluded us from confirming the two potential reinfections detected in the reinfection follow-up group by phylogenetic analysis of paired baseline and follow-up samples, as these samples were not available; nevertheless, it is important to note that in both cases, SVR was confirmed by a negative HCV-RNA test, and that relapse post-SVR is generally rare (< 1%)^31^. Finally, in the clustering analyses performed, a slight overlap existed between the right tail of the recent transmission distribution (Fig. 3a, *same isolate*) and the left tail of the distribution among isolates of the same subtype (Fig. 3b, *same subtype, different isolate*), therefore the selected cut-off value of two might have resulted in the detection of a small fraction of false negatives and false positives. Moreover, as no personal identification was recorded in the HepC*detect* II study, in the four clusters of two sequences involving one participant from each study (representing 16% of all clusters), the fact that both sequences belonged to the same individual identified both inside and outside the prison could not be ruled out. This could have led to a slight overestimation of the proportion of epidemiologically related HCV isolates both in and out of prison.

In conclusion, the HCV reinfection rate is low in people treated with DAAs in Catalan prisons, but it is frequent in people who enter prison, especially in those individuals who show a vulnerable and risky behavioral profile for HCV infection such as PWID. Incarceration may provide a unique opportunity to improve the health of HCV-infected individuals, who often do not attend mainstream healthcare services, including PWID and other marginalized populations, and to prevent the spread of HCV to the community. Nevertheless, this should not detract from the fact that there is a need to strengthen harm reduction and test and treat programs among PWID in a comprehensive manner (both in prisons and in the community) to maintain low HCV prevalence in prisons and achieve HCV elimination.

### Supplementary Information

Supplementary Figure 1.

Supplementary Figure 2.

Supplementary Information 3.

## Data Availability

The datasets generated and/or analysed during the current study are available from the corresponding author on reasonable request. Nucleotide sequences are available GenBank (accession numbers OQ342879-OQ343045).
