# Author Correction: Incidence and molecular epidemiology of hepatitis C virus reinfection in prisons in Catalonia, Spain (Re-HCV study)

**DOI:** 10.1038/s41598-023-44581-x

**Published:** 2023-10-12

**Authors:** Verónica Saludes, Antoni E. Bordoy, Elena Yela, Elisabet Turú, Anna Not, Evelin López-Corbeto, Laia Egea-Cortés, Fernando González-Candelas, Jordi Casabona, Group for the Study and Control of Infectious Diseases in Prison (GRUMIP), Núria Teixidó, Anna Sastre, Ana Ruíz, Carlos Gallego, Carlos Touzón, Concepció Solé, Ramón Planella, Elisa Vaz, Rafael A. Guerrero, Andrés Marco, Elisa Martró

**Affiliations:** 1 Microbiology Department, Northern Metropolitan Clinical Laboratory, Germans Trias i Pujol Research Institute and Hospital (IGTP), Crta. del Canyet S/N, 08916 Badalona, Barcelona Spain; 2 Consortium for Biomedical Research in Epidemiology and Public Health (CIBERESP), Instituto de Salud Carlos III, Madrid, Spain; 3 Brians-1 Prison Health Services, Sant Esteve Ses Rovires, Barcelona, Spain; 4 Prison Health Programme, Catalan Institute of Health (ICS), Barcelona, Spain; 5 Centre for Epidemiological Studies On Sexually Transmitted Infections and HIV/AIDS of Catalonia (CEEISCAT), Public Health Agency of Catalonia (ASPCAT), Badalona, Spain; 6 Joint Research Unit Infection and Public Health FISABIO-University of Valencia I2SysBio, Valencia, Spain; 7 EAPP Sant Esteve Sesrovires-1, Barcelona, Spain; 8 EAPP Sant Esteve Sesrovires-2, Barcelona, Spain; 9 EAPP La Roca del Vallés-1, Barcelona, Spain; 10 EAPP Sant Joan de Vilatorrada, Barcelona, Spain; 11 EAPP Figueres, Girona, Spain; 12 EAPP Lleida, Lleida, Spain; 13 EAPP Tarragona, Tarragona, Spain

Correction to: *Scientific Reports* 10.1038/s41598-023-42701-1, published online 25 September 2023

The Acknowledgments section in the original version of this Article was incomplete.

“The authors specially acknowledge and thank the people in prisons who participated in this study, and the nursing staff at the Catalan prisons. Additionally, we thank the personnel at the reference territorial laboratories (J. Niubó, C. Gutiérrez, A. Bernet, C. González, A. Rando, and D. Pérez) for providing us with clinical samples from study participants. The Re-HCV Study was carried out with the collaboration of the competitive Gilead Scholarship Program for Biomedical Research (grant number GLD18-00052, 2019-2020), but Gilead had no role in the study design, collection, analysis, and interpretation of data, in the writing of the report, or in the decision to publish. A. N. holds a PFIS grant (Ref. FI20/00211, Instituto de Salud Carlos III, Fondo Social Europeo). Finally, the authors thank the CERCA Programme/Generalitat de Catalunya for their support to the Germans Trias i Pujol Research Institute (IGTP).”

now reads:

“The authors specially acknowledge and thank the people in prisons who participated in this study, and the nursing staff at the Catalan prisons. Additionally, we thank the personnel at the reference territorial laboratories (J. Niubo, C. Gutierrez, A. Bernet, C. Gonzalez, A. Rando, and D. Perez) for providing us with clinical samples from study participants. The Re-HCV Study was carried out with the collaboration of the competitive Gilead Scholarship Program for Biomedical Research (grant number GLD18-00052, 2019-2020), and Gilead also supported article processing charges, but had no role in the study design, collection, analysis, and interpretation of data, in the writing of the report, or in the decision to publish. A. N. holds a PFIS grant (Ref. FI20/00211, Instituto de Salud Carlos III, Fondo Social Europeo). Finally, the authors thank the CERCA Programme/Generalitat de Catalunya for their support to the Germans Trias i Pujol Research Institute (IGTP).”

The original Article has been corrected.

