# Supplementary figures and images for: Incidence and molecular epidemiology of hepatitis C virus reinfection in prisons in Catalonia, Spain (Re-HCV study)
Source: Sci Rep. 2023 Sep 25;13:16012. doi: 10.1038/s41598-023-42701-1 (PMC10520040; doi:10.1038/s41598-023-42701-1)

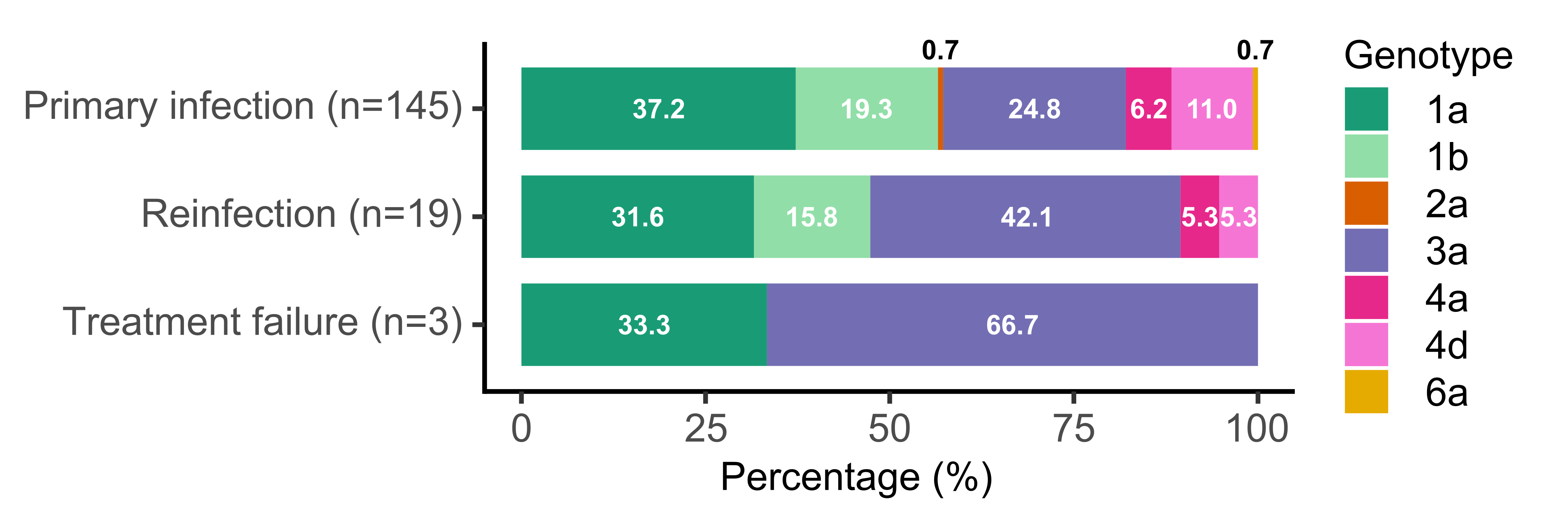

Supplement: Supplementary file 1 — Supplementary Figure 1. [file 41598_2023_42701_MOESM1_ESM.tiff]

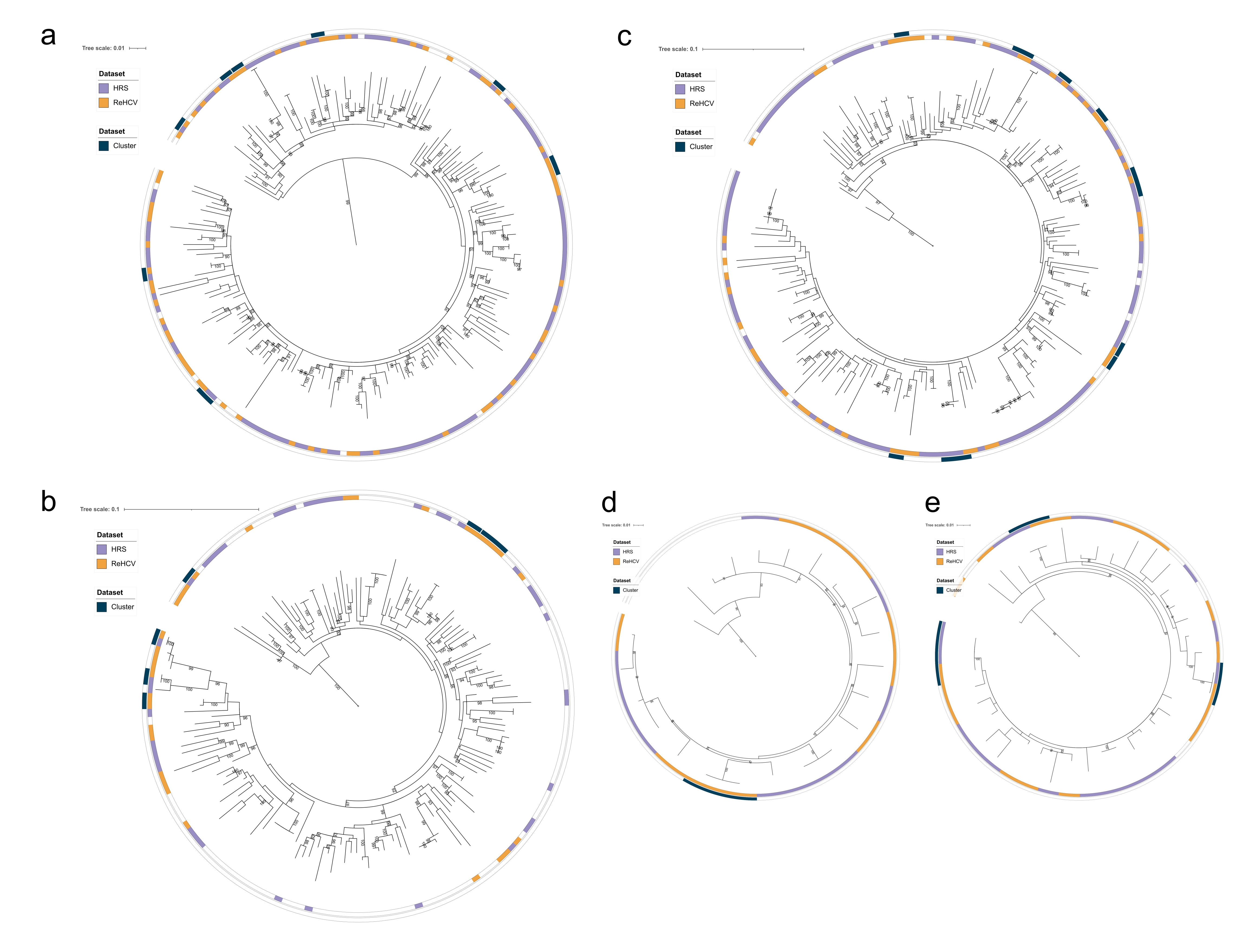

Supplement: Supplementary file 2 — Supplementary Figure 2. [file 41598_2023_42701_MOESM2_ESM.jpg]
