# Supplementary material for: Incidence and molecular epidemiology of hepatitis C virus reinfection in prisons in Catalonia, Spain (Re-HCV study)
Source: Sci Rep. 2023 Sep 25;13:16012. doi: 10.1038/s41598-023-42701-1 (PMC10520040; doi:10.1038/s41598-023-42701-1)
Supplement: Supplementary file 3 — Supplementary Information 3. [file 41598_2023_42701_MOESM3_ESM.docx]

**Incidence and molecular epidemiology of hepatitis C virus reinfection in prisons in**

**Catalonia, Spain (Re-HCV study)**

Verónica Saludes^1,2,^, Antoni E Bordoy^1,^, Elena Yela^3^, Elisabet Turú^4^, Anna Not^1^, Evelin López-Corbeto^2,5^, Laia Egea-Cortés^5^, Fernando González-Candelas^2,6^, Jordi Casabona^2,5^, Group for the Study and Control of Infectious Diseases in Prison (GRUMIP)^4^, Andrés Marco^2,4, †^ and Elisa Martró^1,2,*, †^

^1^ Microbiology Department, Northern Metropolitan Clinical Laboratory, Germans Trias i Pujol Research Institute and Hospital (IGTP), Badalona, Spain.

^2^ Consortium for Biomedical Research in Epidemiology & Public Health (CIBERESP), Instituto de Salud Carlos III, Madrid, Spain.

^3^ Brians-1 Prison Health Services. Sant Esteve Ses Rovires, Barcelona, Spain.

^4^ Prison Health Programme, Catalan Institute of Health (ICS). Barcelona.

^5^ Centre for Epidemiological Studies on Sexually Transmitted Infections and HIV/AIDS of Catalonia (CEEISCAT), Public Health Agency of Catalonia (ASPCAT), Badalona, Spain.

^6^ Joint Research Unit Infection and Public Health FISABIO-University of Valencia I^2^SysBio, Valencia, Spain.

^♦^These authors share first position.

^†^These authors share senior authorship.

***Corresponding author:** Elisa Martró. Microbiology Department, Hospital Universitari Germans Trias i Pujol, Crta. del Canyet s/n, 08916 Badalona (Barcelona), Spain. E-mail: emartro@igtp.cat; Phone: +34 934978894. Fax: +34 934978895.

**Supplementary material**

**METHODS**

**HCV sequencing and phylogenetic analyses.**

*Total RNA extraction from plasma.* RNA was automatically extracted from 250-800 μL of plasma using the Abbott m2000*sp* platform (Abbott Molecular), according to the manufacturer's instructions.

*Retrotranscription.* HCV-RNA was retrotranscribed using random hexamers, in order to prevent any bias during the reaction, and Superscript IV reverse transcriptase (Thermo Fisher Scientific), as previously described [1].

*PCR amplification of the HCV NS5B region.* A 389-bp fragment corresponding to nucleotides 8250-8638 of the NS5B region of the HCV H77 reference genome was amplified with the high-fidelity Velocity DNA polymerase (Bioline), as previously described [2]. Amplicons were purified using Agencourt Ampure XP beads (Beckman-Coulter).

*Sanger sequencing of NS5B amplicons.* Bidirectional Sanger sequencing was performed using the previous PCR primers. Sequence reads were assembled with the Staden Package and manually edited to generate the consensus sequences. Nucleotide sequences were submitted to GenBank (accession numbers OQ342879-OQ343045).

*Phylogenetic analysis.* Sequences obtained from the study participants and the external population (HepC*detect* II study) were aligned with other sequences circulating in Spain (**Supplementary Table 1**) and reference sequences from all confirmed HCV genotypes and subtypes (available from: https://ictv.global/sg_wiki/flaviviridae/hepacivirus/hcv_files) using mafft [3]. The resulting multiple alignment was manually trimmed to a final length of 328-bp and used to find the evolutionary model that best fitted the data and to reconstruct a Maximum Likelihood phylogenetic tree with IQ-TREE 2 [4]. The empirical support for the nodes of the phylogenetic tree was assessed by ultrafast bootstrap analysis implemented in IQ-TREE (1000 replicates). From this tree, HCV isolates were classified at genotype and subtype levels, and epidemiological relationships were assessed. Regarding the latter, clusters of recent transmission involving HCV isolates in Catalan prisons were determined according to a threshold based on the distribution of pairwise single nucleotide polymorphisms (SNPs) between isolates of the same subtype. Since pairwise SNPs in a given period of time follow a Poisson distribution, the mode of the empirical distribution was fitted in RStudio v4.1.0 using locmodes function of the multimode package. The mode was then used to calculate the first natural number that resulted in a value of the cumulative distribution function >95%, which was established as the cut-off value. This threshold was compared to the expected number of SNPs in a period of five years (which was the maximum timespan separating the collection dates of samples from the Re-HCV and HepC*detect* II studies) calculated from the mean of the nucleotide substitution rate of the NS5B region across genotypes 1a, 1b, 3a and 4 (1.43×10^-3^ substitutions/site/year [5–7]. Monophyletic clusters containing at least one sample from the Re-HCV study, fulfilling the established pairwise SNP cut-off and supported by a bootstrap value ≥90% were identified.

**REFERENCES**

1. Saludes, V. *et al*. Relevance of baseline viral genetic heterogeneity and host factors for treatment outcome prediction in hepatitis C virus 1b-infected patients. *PLoS One* **8**, e72600 (2013).

2. Antuori, A. *et al*. Characterization of acute HCV infection and transmission networks in people who currently inject drugs in Catalonia: usefulness of dried blood spots. *Hepatology* **74**, 591–606 (2021).

3. Katoh, K. & Standley, D. M. MAFFT multiple sequence alignment software version 7: improvements in performance and usability. *Mol. Biol. Evol*. **30**, 772–780 (2013).

4. Minh, B. Q. *et al*. IQ-TREE 2: new models and efficient methods for phylogenetic inference in the genomic era. *Mol. Biol. Evol*. **37**, 1530–1534 (2020).

5. Di Lello, F. A. *et al*. Hepatitis C virus genotype 4 in Southern and Central Spain does not originate from recent foreign migration waves. *J. Med. Virol*. **85**, 1734–1740 (2013).

6. Romano, C. M. *et al*. Social networks shape the transmission dynamics of hepatitis C virus. *PLoS One* **5**, e11170 (2010).

7. Yuan, M., Lu, T., Li, C., & Lu L. The evolutionary rates of HCV estimated with subtype 1a and 1b sequences over the ORF length and in different genomic regions. *PLoS One* **8**, e64698 (2013).

**CONSORTIUM NAME**

Consortium name for the Group for the Study and Control of Infectious Diseases in Prison (GRUMIP):

Núria Teixidó^4,5^, Anna Sastre^4,6^, Ana Ruíz^4,6^, Carlos Gallego^4,7^, Carlos Touzón^4,8^, Concepció Solé^4,9^, Ramón Planella^4,10^, Elisa Vaz ^4,11^, Rafael A. Guerrero^4^.

^4^ Prison Health Programme, Catalan Institute of Health (ICS), Barcelona, Spain.

^5^ EAPP Sant Esteve Sesrovires-1, Barcelona, Spain.

^6^ EAPP Sant Esteve Sesrovires-2, Barcelona, Spain.

^7^ EAPP La Roca del Vallés-1, Barcelona, Spain.

^8^ EAPP Sant Joan de Vilatorrada. Barcelona. Spain.

^9^ EAPP Figueres, Girona, Spain.

^10^ EAPP Lleida, Lleida, Spain.

^11^ EAPP Tarragona, Tarragona, Spain.

**SUPPLEMENTARY FIGURE LEGENDS**

**Supplementary Figure 1. HCV genotypes among viremic people in prison detected at incarceration (viremic group) according to sequencing and phylogenetic analysis of the NS5B region.**

**Supplementary Figure 2. Maximum likelihood phylogenetic trees of selected HCV subtypes.** Nucleotide substitution model GTR + I + G4. (a) subtype 1a, (b) subtype 1b, (c) subtype 3a, (d) subtype 4a, and (e) subtype 4b. The inner annotation ring indicates samples belonging to the ReHCV study (orange) or collected from individuals attending harm reduction services (HRS, purple). Clusters supported by a monophyletic origin, a bootstrap value ≥90% and a maximum of three SNPs (NS5B region) between pairs of isolates are marked in the outer annotation ring. The scale bar represents number of substitutions per nucleotide position.

**SUPPLEMENTARY TaBLE**

**Supplementary Table 1. HCV sequences circulating in Spain used for phylogenetic analysis.**

|  |  |
| --- | --- |
| **GenBank accession number** | **Source** |
| AJ880694.1-AJ880757.1 | Bracho MA, Gosalbes MJ, Blasco D, Moya A, González-Candelas F. Molecular epidemiology of a hepatitis C virus outbreak in a hemodialysis unit. J Clin Microbiol. 2005;43(6):2750-5. |
| LN845875.1 - LN845883.1 | Saludes V, Quer J, Gregori J, Bascuñana E, García-Cehic D, Esteban JI et al. Identification of hepatitis C virus genotype 3 by a commercial assay challenged by natural polymorphisms detected in Spain from patients with diverse origins. J Clin Virol. 2016;78:14-9. |
| EF608894.1 - EF608922.1, EF608925.1 | Massaguer A, Ramírez S, Carrión JA, González P, Sánchez-Tapias JM, Forns X. Evolution of the NS3 and NS5B regions of the hepatitis C virus during disease recurrence after liver transplantation. Am J Transplant. 2007;7(9):2172-9. |
|  |  |
